# Preparation and Kinetic Studies of Cross-Linked Chitosan Beads Using Dual Crosslinkers of Tripolyphosphate and Epichlorohydrin for Adsorption of Methyl Orange

**DOI:** 10.1155/2021/6648457

**Published:** 2021-02-17

**Authors:** Akhmad Sabarudin, Armeida D. R. Madjid

**Affiliations:** ^1^Department of Chemistry, Faculty of Science, Brawijaya University, Malang 65145, Indonesia; ^2^Department of Chemistry, Faculty of Science and Technology, State Islamic University of Maulana Malik Ibrahim, Malang 65145, Indonesia

## Abstract

Preparation of cross-linked chitosan beads using dual crosslinkers of tripolyphosphate (TPP) and epichlorohydrin (ECH) for the adsorption and kinetic studies of methyl orange (MO) had been carried out. FTIR spectra showed that TPP could act as the protecting agent of the NH_2_ group of chitosan and ECH reacted with the primary hydroxyl group of chitosan. Various concentrations of TPP, ECH, and immersing time in the TPP solution for bead formation were studied. The effect of pH and kinetics of adsorption were investigated to define the mechanism of adsorption and rate-limiting step. As a result, pH 3, 10% (w/v) TPP, 5% (v/v) ECH, and 12 h immersing time in TPP were selected as the optimum conditions for preparing the beads as indicated by the highest adsorption amount of MO. The cross-linked chitosan beads' adsorption capacity for MO under optimum condition was found to be 79.55 mg/g with the adsorption rate constant (*k*) of 1.29 × 10^−3^/min. Furthermore, it was found that a low concentration of ECH could maintain the stability of chitosan in acidic conditions, whereas the concentration of TPP and immersing time controlled pore size and morphology of chitosan beads. The mechanism of adsorption of MO was controlled by the pore and rigidity of cross-linked chitosan beads. Bulk diffusion acted as a rate-limiting step, and a high concentration of MO inhibited diffusion and adsorption itself.

## 1. Introduction

Methyl orange (MO) is an azo dye widely used as a coloring agent and indicator in the titration method. Methyl orange is not toxic, but it can be hypersensitivity and allergy triggers [1]. Dyes reduce sunlight penetration in water, disturb the photosynthesis process, and lessen oxygen content. The most popular approaches for dye removal are photocatalytic degradation [2, 3] and adsorption [4, 5] methods. Although photodegradation methods using nanostructured materials [6] and nanocomposites [7] have developed rapidly, however, adsorption method remains in popularity due to its benefit that it can prevent the production of other toxic substances [8–10]. Furthermore, the toxicity of dyes will not affect the adsorption process. The adsorption method is a simple operation and easy to modify adsorbents to enhance their adsorption capacity [11]. Some materials frequently used as the adsorbent for metallic and nonmetallic compounds are natural biomass, which includes cellulose, rice husk, and other bioadsorbents [12, 13], activated and modified activated carbons [14, 15], and waste materials [16, 17].

Chitosan, derived from the shells of shrimp and other sea crustaceans, is the natural adsorbent material classified with high adsorption capacity and available abundantly [11, 18]. Chitosan can be modified physically or chemically to improve its stability. Physical modification of chitosan into the bead by employing tripolyphosphate (TPP) as the ionic crosslinker resulted in more rigid and stable drying processes. These beads would shrink, harden, and would not turn into flake [19]. However, chitosan is unstable in acids indicated by a complete solubility in acetic acid and partially dissolve in concentrated nitric acid and hydrochloric acid. For solving this problem, chitosan must be chemically cross-linked to be applied in a wide pH range [20–22], and it can be further functionalized with aliphatic [23, 24] and aromatic [25–27] moieties for more specific adsorption-desorption applications. In comparison to ethylene glycol diglycidyl ether (EGDE) [21–27], epichlorohydrin (ECH) seems to be the best option choice as a chemically bonding crosslinker, which is more appropriate for a large molecule like methyl orange (MO) [28].

Chitosan, a deacetylated form of chitin, has the functional group amine (−NH_2_) and hydroxyl (OH^−^) as affinity centers [29]. In the acidic region, the amine (NH_2_) group will be protonated into NH_3_^+^ and interact with azo dyes. As a crosslinker, epichlorohydrin (ECH) is easily reacted with the NH_2_ group, resulting in lower adsorption capacity of chitosan [30, 31]. Tripolyphospate (TPP) can be applied as the double agents for beads formation and NH_2_ group protector. For the modification of chitosan, TPP must be reacted first, followed by adding ECH for a cross-linking reaction via the primary hydroxyl group of chitosan. The addition of concentrate ECH [32, 33] or base ECH solution [1, 19] should be avoided because those would ruin beads and loose TPP from chitosan. In base condition, chitosan is still in the deprotonated form, and TPP would compete with hydroxyl (OH^−^) to link with chitosan [34].

Adsorption is a process of concentration migration from one phase to another. There are many approaches and models for the adsorption evaluation process, such as equilibrium isotherm models, kinetics models, or thermochemistry models [5, 35]. Each method used its assumption to interpret the relationship between time and the reduction of adsorbate concentration, which migrates into the adsorbent. The assumption was mostly dependent on adsorbent capacity or the ability of the adsorbent to trap adsorbate inside. Kinetic models are methods frequently used to evaluate the adsorption process. Pseudo-first-order (Lagergren models), pseudo-second-order (Ho and McKay Model), and Intraparticle Diffusion (Weber and Morris Model) were several common kinetic models [18, 19].

**Figure 1:**
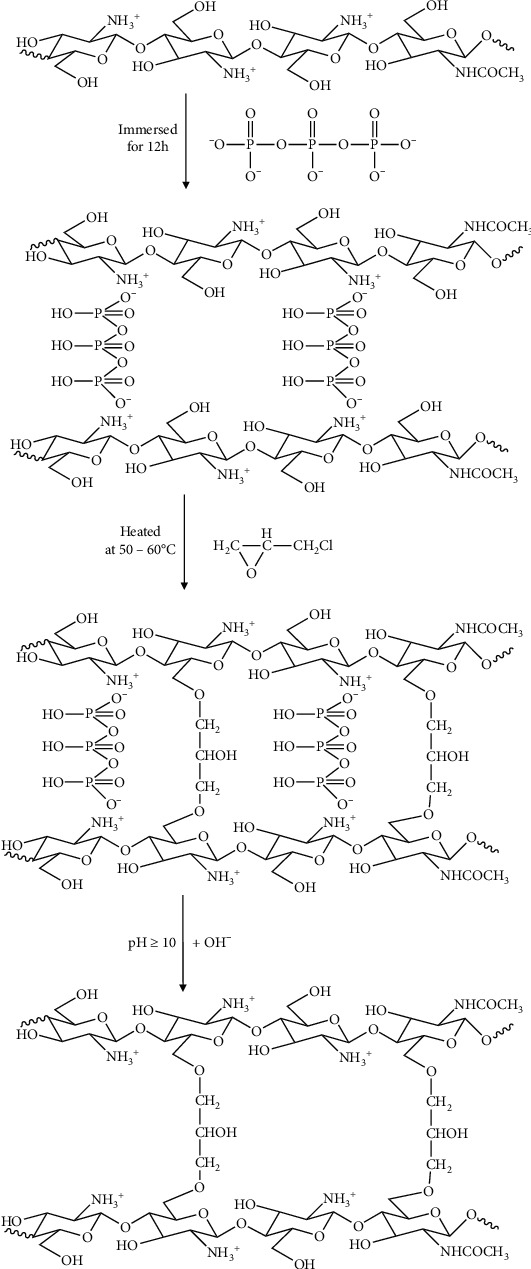
Reaction steps for the preparation of cross-linked chitosan beads.

Usually, ECH as a crosslinker is reacted with the amine group of chitosan resulting in a considerable reduction of chitosan's adsorption capacity. Accordingly, in this work, a different strategy using a dual crosslinker as shown in Figure 1 was applied in regard to the adsorption capacity of chitosan. Firstly, chitosan was modified by adding TPP as an ionic crosslinker to form spherical beads and to protect its amine group. Secondly, the primary hydroxyl group of chitosan beads was reacted with ECH via the opening-ring reaction of its epoxide group. In the final step, TPP was released by conditioning the beads at pH ≥10, resulting in an un-protected amine (NH_2_) group of chitosan, which provides high sorption capacity and is a very reactive adsorbent's active site. The produced cross-linked chitosan beads were characterized using FTIR and SEM. Adsorption evaluation was carried out through the removal of methyl orange (MO) using the batch method. The adsorption mechanism was investigated by varying the pH of the MO solution and contact time/adsorption time. Furthermore, the kinetics models were studied using the adsorption capacity active site adsorbent model and reduction of adsorbate concentration in the bulk solution (fractional time method).

## 2. Materials and Methods

### 2.1. Materials

Chitosan, epichlorohydrin (ECH, 99%), sodium tripolyphospate (TPP), methyl orange (MO), hydrochloric acid (HCl, 37%), and sodium hydroxide (NaOH) were purchased from Sigma-Aldrich (Singapore). All materials were of analytical grades except sodium TPP (Technical Grade, 85%).

### 2.2. Instrumentations

The amount of MO adsorbed was measured using a Genesys 10S UV-Vis Spectrophotometer at the wavelength of 464 nm. SEM analysis for morphology assessment of the resulted cross-linked chitosan bead was performed using Hitachi TM3000. The Shimadzu FTIR 8400 was employed for functional group characterization.

### 2.3. Preparation of Cross-Linked Chitosan Beads

One Gram of chitosan was dissolved in 100 ml acetic acid 5% (v/v) and left overnight. Then, 5 ml chitosan solution dropped into TPP (1%, 5%, 10%, and 15% w/v) using a syringe and immersed at various time conditions (1, 3, 6, 12, and 24 h) and filtered. Chitosan beads were added to ECH solution in distilled water (1%, 2.5%, and 5% v/v) and heated for 2 h at 50–60°C while stirring. After the cross-linking process, NaOH was added to raise pH ≥ 10 and left to stand for an hour. Then, cross-linked chitosan beads were filtered, washed with distilled water and ethanol, and dried at 105°C until constant weight. FTIR was used to confirm the success of the cross-linked chitosan bead preparation by identifying the specific binding group of chitosan with TPP and ECH.

### 2.4. Adsorption of Methyl Orange (MO)

0.02 gram of cross-linked chitosan beads was used for the adsorption of methyl orange (50 mL and 20 ppm) by a batchwise method for 2 h and a shaking rate of 100 rpm. The effect of pH was studied by varying the acidity of methyl orange in the range of pH 2–8. The pHs were adjusted by the addition of HCl or NaOH solution. For adsorption kinetics investigation, 100 mL of methyl orange solution was adjusted to optimum pH and then stirred for 24 h. The stirring was halted at an appropriate time interval, and several aliquots were taken from the supernatant using a micropipette. An aliquot was adjusted to pH 7 then diluted to adequate volume for UV measurement at *λ* 464 nm. An amount of methyl orange adsorbed was calculated according to the following equation:

(1) $$\begin{array}{c} q=\;\frac{\left(C_{0}-C_{e}\right)V}{m}, \end{array}$$

where *q* is the amount of methyl orange adsorbed by cross-linked chitosan beads (mg/g) and C_0_ and *C*_*e*_ are the methyl orange concentrations (mg/L), respectively. *V* is the volume of the solution (*L*), and *m* is the mass of the adsorbent used.

## 3. Results and Discussion

### 3.1. Preparation and Characterization of the Cross-Linked Chitosan Bead

**Figure 2:**
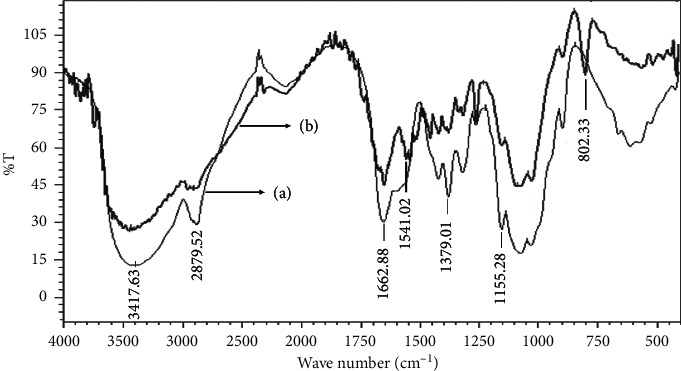
IR spectra of chitosan (a) and cross-linked chitosan beads (b).

Cross-linked chitosan beads were prepared by dropping chitosan solution into TPP to protect the NH_2_ group of chitosan and to form spherical beads. Then, ECH was reacted with the primary hydroxyl group of chitosan to improve the chemical stability of chitosan, followed by removing TPP from the NH_2_ group chitosan to improve the adsorption capacity of the resulted beads. To confirm the successful reactions, the products were characterized using FTIR, and the peak profiles were compared between the original chitosan and cross-linked chitosan beads, as shown in Figure 2. It was found that there was a declined intensity in 3400 cm^−1^, confirming the interaction of the OH^−^ group in chitosan with ECH. Additionally, there was a declined intensity in 1200–1250 cm^−1^, indicating ECH's interaction with the NH_2_ group in chitosan. As the purpose of adding TPP in the preparation step was to prevent interaction of NH_2_ with ECH, however, FTIR confirmed that a part of NH_2_ is still linked with ECH. It means that TPP is unable to protect the NH_2_ group of chitosan completely, although some peaks at 1650 cm^−1^, 1541 cm^−1^, and 1151 cm^−1^ confirmed the interaction between NH_2_ of chitosan and TPP [36].

**Figure 3:**
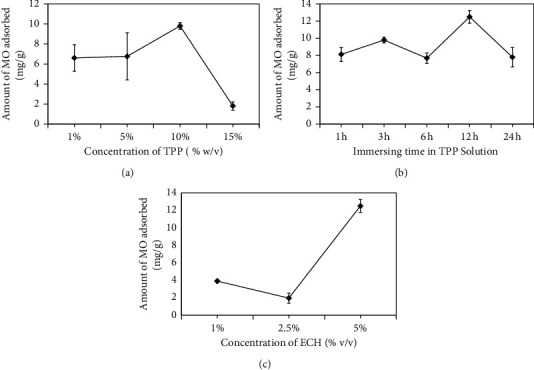
Effect of TPP concentration at conditions of 12 h immersing time and ECH 5% (v/v) (a), immersing time of chitosan in TPP solution at conditions of TPP 10% (w/v) and ECH 5% (v/v) (b), and concentration of ECH at conditions of 12 h immersing time in TPP 10% (w/v) (c) for the preparation of the cross-linked chitosan beads. Sample: 50 mL methyl orange 20 ppm; cross-linked chitosan beads mass: 0.02 g; stirring rate = 100 rpm; pH 4.

**Figure 4:**
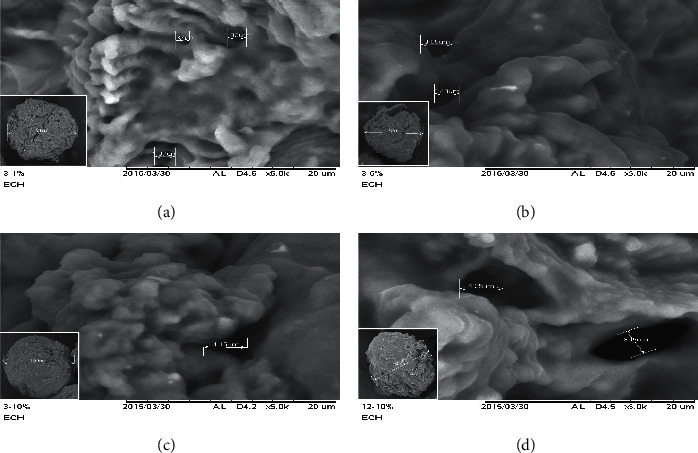
SEM images of cross-linked chitosan beads using 5% (v/v) ECH immersed in TPP 1% for 3 h (a), TPP 5% for 3 h (b), TPP 10% for 3 h (c), and TPP 10% for 12 h (d) with magnification of 5000x. Inset pictures are SEM images of the beads with a magnification of 100x.

For the preparation of cross-linked chitosan beads, several parameters involving TPP concentration, immersing time of the bead in TPP, and ECH concentration were optimized. The effects of those parameters were evaluated by MO's adsorption onto cross-linked chitosan beads, as shown in Figure 3. It was found that TPP concentration affected effective porosity, whereas immersing time in TPP modified pore morphology as confirmed by the SEM micrograph (Figure 4).

At similar ECH concentrations (5%), it was found that a higher concentration of TPP and longer immersing time resulted in smaller pore sizes of beads and smoother surface morphology. Intermolecular and intramolecular bonds seem responsible for the link between TPP and chitosan. At a higher concentration of TPP, pore formation (intermolecular bond) occurred more effectively, whereas the intramolecular bond started forming (Figures 4(a)–4(c)). Intramolecular links caused beads to become more rigid and harder. At a longer immersing time in TPP, beads' morphology was smoother due to increment intramolecular bond formed (Figures 4(c)–4(d)). An appropriate proportion of intermolecular and intramolecular bonds in beads affected MO's adsorption onto cross-linked chitosan beads. ECH, as a crosslinker, is aimed to improve the chemical stability of chitosan beads in acid. So, the adsorption can be performed in acid conditions without any deterioration. From these optimization steps, cross-linked chitosan beads made by 12 h immersing in TPP 10% (w/v) with ECH 5% (v/v) showed the best results as indicated by the highest amount of the adsorbed MO.

### 3.2. Adsorption of MO onto the Cross-Linked Chitosan Bead

**Figure 5:**
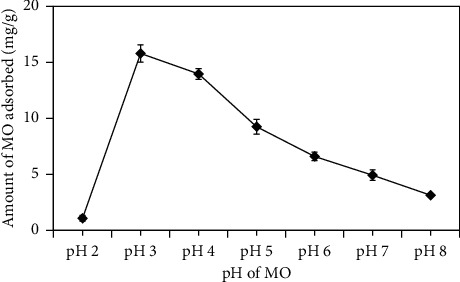
Effects of pH on the adsorption of MO using cross-linked chitosan beads. The bead crosslinkers are ECH 5% immersed in TPP 10% for 12 h; sample: 50 mL methyl orange 20 ppm; cross-linked chitosan bead's mass: 0.02 g; and stirring rate = 100 rpm.

The acidity (pH) strongly affects the adsorption of MO onto cross-linked chitosan beads.  Figure 5 exhibits the effect of pH toward the amount of MO adsorbed from pH 2 to 8. The highest amount of MO adsorbed was achieved at pH 3. Then, along with the increment of pH, the amount of MO adsorbed decreased. pH affected the form of adsorbate (MO) and the protonation of the NH_2_ group of chitosan. Methyl orange/MO (pKa 3.4) exists in the form of a zwitterion in acidic conditions and an anion in the base solution [37], whereas the NH_2_ group of chitosan (pKa 6.5) protonates into –NH_3_^+^ at the acidic condition [21–23].

**Figure 6:**
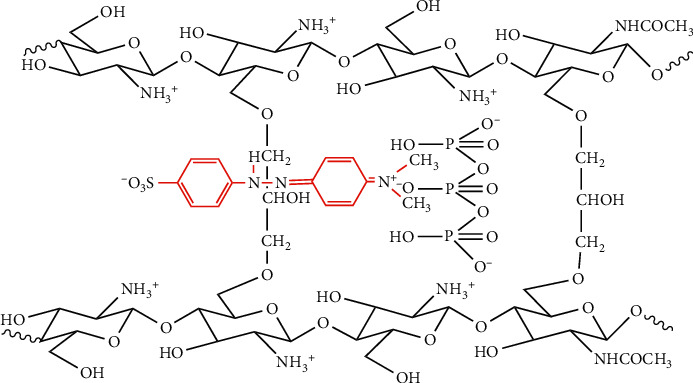
Adsorption mechanism of methyl orange onto cross-linked chitosan beads.

Because MO forms zwitterions and –NH_2_ of chitosan were protonated into –NH_3_^+^ at pH 3, the repulsion between positive charge in MO and NH_3_^+^ occurred, resulting in low adsorption amounts of MO. However, as shown in Figure 5, the highest amount of MO was achieved at this pH. This condition revealed the role of TPP in adsorption diffusion. At pH 3, TPP exists in the form of H_2_P_3_O_10_^3−^ where its two negative charges interact with protonated chitosan while the remaining one reacts with positively charged MO. This adsorption mechanism is illustrated in Figure 6. Modification of chitosan using ECH and TPP can extend chitosan application in the broad range of pH conditions. It was indicated by the cross-linked chitosan beads' excellent stability at a range of the examined pH (2–8).

**Figure 7:**
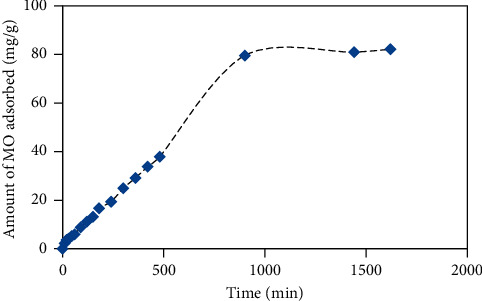
Effect of contact time on the adsorption of methyl orange onto cross-linked chitosan beads. Sample: 100 mL methyl orange 20 ppm; chitosan bead's mass: 0.02 g; stirring rate: 100 rpm.

The cross-linked chitosan bead's adsorption capacity was examined by equilibrating an appropriate amount of the bead with the excess amount of MO solution under optimum conditions. As shown in Figure 7, it was found that the adsorption capacity of the cross-linked chitosan bead towards MO was estimated to be 79.55 mg/g. Besides the simple synthesis procedure, compared to other adsorbents for the removal of MO, the cross-linked chitosan bead prepared in this work exhibits comparable and/or higher adsorption capacity as shown in Table 1. Accordingly, these beads provide the good potential to be used as an adsorbent for the removal of MO and other dyes in the environmental samples.

### 3.3. Adsorption Kinetics of MO onto the Cross-Linked Chitosan Bead

**Figure 8:**
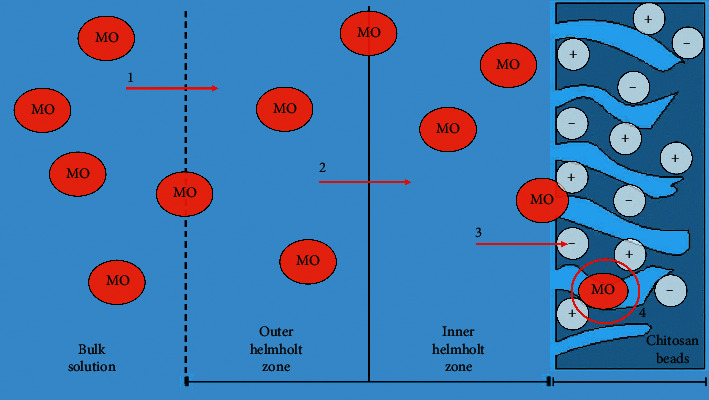
Diffusion steps of methyl orange from the bulk solution into cross-linked chitosan beads (1- bulk diffusion, 2- film diffusion, 3- intraparticle diffusion, and 4- adsorption on chitosan beads).

Adsorption steps of MO onto the cross-linked chitosan bead were considered to involve four steps, as illustrated in Figure 8:

- Bulk diffusion, mass transfer of adsorbate from bulk solution into the outer Helmholtz plane
- Film diffusion, adsorbate shift from the outer Helmholtz plane into the inner Helmholtz plane
- Intraparticle diffusion, adsorbate transfer into the affinity site of the adsorbent
- Adsorption process, adsorbate trap in the adsorbent chemically or physically

One of these steps acts as a rate-limiting step. The reaction rate of adsorption was investigated by using pseudo-first-order, pseudo-second-order, and interparticle diffusion models. Pseudo-first-order and pseudo-second-order models were based on the active site of an adsorbent. Intraparticle diffusion models reconstructed mass transfer of the adsorbate from the bulk solution into the adsorbent. The adsorption process is controlled by the adsorbent's capacity and the concentration of adsorbate. The adsorbate concentration is the driving force of mass transfer from the bulk solution into an adsorbent's inner. Based on the adsorbate concentration, the order of reaction should be determined firstly by calculating the reaction rate. The order reaction was determined using a fractional time method.

#### 3.3.1. Active Site Capacity Kinetics Model

**Figure 9:**
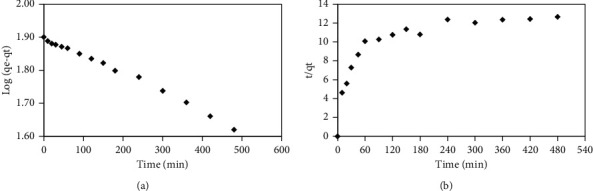
Pseudo-first-order plot (a) and pseudo-second-order plot (b) for the adsorption of MO onto cross-linked chitosan beads.

The pseudo-first-order kinetics model is given by the following equation:

(2) $$\begin{array}{c} \log\left(\frac{q_{e}}{q_{e}-q_{t}}\right)=\frac{k_{1}}{2.303}t, \end{array}$$

where *q*_*e*_ and *q*_*t*_ are the amount of methyl orange adsorbed in equilibrium and at *t* and *k*_1_ is a rate constant of the pseudo-first-order kinetic model (min^−1^). The straight-line plot log *(q*_*e*_*and q*_*t*_) against *t* was used to determine *k*_*1*_ and *r*, correlation coefficient, which can be seen in Figure 9(a).

The pseudo-second-order kinetic model is given in the following equation:

(3) $$\begin{array}{c} \frac{1}{q_{e}-q_{t}}=\frac{1}{q_{e}}+k_{2}t, \end{array}$$

where *q*_*e*_ and *q*_*t*_ are the amount of methyl orange adsorbed in equilibrium and at *t* and *k*_2_ is a rate constant of the pseudo-second-order kinetic model (mg gram^−1^ min^−1^). The straight-line plot *t*/*q*_*t*_ against *t* was used to determine *k*_*2*_ and *r*, correlation coefficient, which can be seen in Figure 9(b). The active site capacity model showed that kinetic adsorption of methyl orange onto cross-link chitosan beads fitted to pseudo-first-order (*r* = 0.994) than pseudo-second-order (*r* = 0.645). Irreversible adsorption occurred because methyl orange (MO) was trapped in the pore of chitosan beads.

#### 3.3.2. Intraparticle Diffusion Kinetics Model

**Figure 10:**
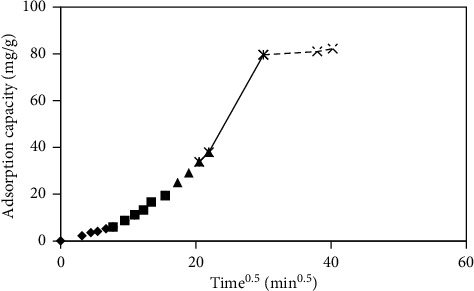
Intraparticle diffusion plot for the adsorption of methyl orange onto cross-linked chitosan beads.

The following equation gives the intraparticle diffusion kinetic model:where *q* refers to the amount of methyl orange adsorbed at *t* and *k*_*i*_ is a diffusion rate (mg/g min0.5). There were five linearity portions in intraparticle diffusion kinetics models. It indicated five steps that occurred in the adsorption process (Figure 10). The first step (ki_1_-♦) referred to bulk diffusion, ki_2_ (■) related to film diffusion, ki_3_ (▲) referred to intraparticle diffusion, ki_4_ ( ) associated with the adsorption step, and ki_5_ ( ) referred to the equilibrium step. At the end, diffusion was getting slower due to the low concentration of methyl orange left in the solution. Figure 10 exhibits that ki_1_ was the slowest rate reaction. It means that the rate-limiting process was bulk diffusion.

(4) $$\begin{array}{c} q_{t}=\;k_{i}t^{0.5}. \end{array}$$

#### 3.3.3. Adsorbate Concentration Kinetics Model

**Figure 11:**
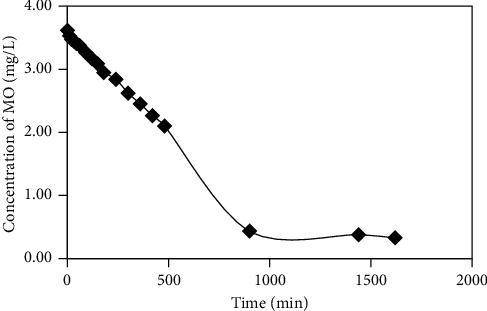
Effect of contact time on reducing adsorbate concentration during the adsorption of methyl orange onto cross-linked chitosan beads.

Adsorbate concentration in the solution decreased linearly at a longer contact time with adsorbent and tended to constant after equilibrium was reached as shown in Figure 11. It implies that the reduction of adsorbate concentration is proportional to the adsorption process. The fractional time method was chosen to determine the order reaction that focuses on proving that the reaction proceeds as first order or not. The order of reaction relates to the adsorption mechanism.

Fractional time is a time when concentration C ([C]) becomes *α* times from initial concentration [C]_0_, and the range of *α* is 0 to 1. We assumed that the reaction was irreversible, so if *n* (reaction order) = 1, *tα* is given by the following equation [47]:

(5) $$\begin{array}{c} t_{\alpha }=\;-\frac{\ln\;\alpha }{k_{a}}. \end{array}$$

For *n* ≠ 1, *t*_*α*_ is given by the following equation:

(6) $$\begin{array}{c} \;t_{\alpha }=\frac{\alpha^{1-n}-1}{\left(n-1\right)\;k_{a}\;{\left[C\right]}_{0}^{n-1}\;}. \end{array}$$

Equation (6) can be converted into a linear form as follows:

(7) $$\begin{array}{c} \ln\;t_{\alpha }=\;\frac{\ln\;\alpha^{n-1}-\;1}{\left(n-1\right)k_{a}}-\;\left(n-1\right)\ln\;{\left[C\right]}_{0}. \end{array}$$

**Figure 12:**
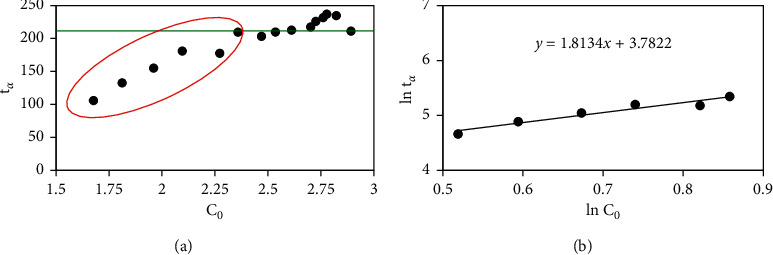
Fractional time models for *n* = 1 (a) and fractional time models for *n* ≠ 1 (b) for the adsorption of methyl orange onto cross-linked chitosan beads.

Then, if we make a relation graph between *t*_*α*_ (min) with [c]_0_ (ppm) (Figure 12(a)), it can be seen that the fractional time of each methyl orange concentration is not constant. It means that a reaction order (*n*) ≠ 1. Figure 12(a) showed two regions of kinetics which fit and unfit to the first-order reaction. The circled area was unfitted to the first-order reaction. So, recalculation of the reaction order with fractional times for the *n* ≠ 1 equation must be performed.  Figure 12(b) shows fractional time calculation for *n* ≠ . From a straight-line plot between ln *t*_*α*_ vs. ln *C*_0_, it was found (slope of plot equal with −(n−1) that the order of the reaction was -0.8134 ≈ −1. This result indicated a higher concentration of methyl orange (MO) would inhibit diffusion or adsorption itself.

All calculations dealing with kinetic adsorption models are summarized in Table 2. The investigation of each kinetic model showed that methyl orange (MO) adsorption onto cross-linked chitosan beads is more fit for the pseudo-first-order model than the pseudo-second-order model. Pseudo-first-order kinetic models plotted with excellent linearity (*r* = 0.994), and an amount of *q*_e(exp)_ closed to *q*_e(theory)_. Bulk diffusion was a rate-limiting step, but methyl orange concentration could inhibit diffusion and adsorption itself.

## 4. Conclusions

The new preparation method of a cross-linked chitosan bead has been designed by adding a protective group of tripolyphospate (TPP) to prevent chemical binding between NH_2_ of chitosan and epichlorohydrin (ECH), which could reduce the adsorption capacity of the bead towards MO. After removing the protective group from the bead, NH_2_ of chitosan could freely attract the anionic dye methyl orange, resulting in an adsorption capacity of 79.55 mg/g. The adsorption mechanism of MO onto the cross-linked chitosan bead occurred physically, which was controlled by the pore and rigidity of cross-linked chitosan beads.

## Figures and Tables

**Table 1:** Adsorption capacities of several adsorbents for the methyl orange adsorption.

Adsorbent	Adsorption capacity (mg/g)	Reference
Hollow molybdenum disulfide microspheres	41.5	[37]
Polyaniline nano-adsorbent	75.9	[38]
Goethite impregnated with chitosan beads	84.0	[39]
*γ*-Fe_2_O_3_/SiO_2_/chitosan composite	34.3	[40]
Chitosan/alumina composite	35.3	[41]
Hyper-cross-linked polymer	76.9	[42]
Polyaniline on a glass plate	93.0	[43]
Magnetic iron oxide/carbon nanocomposites	72.7	[44]
Polyacrylonitrile-coated kapok hollow microtubes	34.7	[45]
Magnetic chitosan enwrapping nanosized *γ*-Fe_2_O_3_ and multiwalled carbon nanotubes	66.1	[46]
Dual crosslinker-TPP/ECH-chitosan bead	79.5	This work

**Table 2:** Summary of the kinetic adsorption calculation.

Kinetics model	*q*_e (calc)_	*k*	*r* ^2^
*Active site capacity*			
Pseudo 1^st^ order	7.928 × 10^1^ mg/g	1.29 × 10^−3^/min	0.994
Pseudo 2^st^ order	7.468 mg/g	2.328 × 10^−5^ g/mg.min	0.645

*Intraparticle diffusion*			
*K*_i1_		0.777 mg/g.min^0.5^	0.994
*K*_i2_		1.782 mg/g.min^0.5^	0.990
*K*_i3_		2.865 mg/g.min^0.5^	0.999
*K*_i4_		4.930 mg/g.min^0.5^	0.996
*K*_i5_		0.223 mg/g.min^0.5^	0.934

*Fractional time*			
*n* ≠ 1		9.72 × 10^−2^ mg/L.min	0.933
*n* = 1		1.03 × 10^−3^/min	

## Data Availability

All data used to support the findings of this study are available from the corresponding author upon request.
